# Tigecycline in the treatment of fulminant *Mycoplasma pneumoniae* pneumonia non-responsive to azithromycin and fluoroquinolone: A case report

**DOI:** 10.1097/MD.0000000000021128

**Published:** 2020-07-10

**Authors:** Xin Yuan, Jie Liu, Changqing Bai, Wenkai Niu, Jingjing Wang, Puyuan Li, Huiying Liu, Yuan Luo

**Affiliations:** a Department of Respiratory and Critical Care Medicine, the Fifth Medical Centre of Chinese PLA General Hospital, Beijing; b Department of Emergency, Hainan Hospital of Chinese PLA General Hospital, Sanya; c Institute of Pharmacology and Toxicology, Academy of Military Medical Sciences, Beijing, China.

**Keywords:** azithromycin, drug resistance, fluoroquinolones, *Mycoplasma pneumoniae*, tigecycline

## Abstract

**Rationale:**

Fulminant macrolide-resistant *Mycoplasma pneumonia*e pneumonia (MPP) has seldom been reported, and cases of MPP usually show rapid improvement after fluoroquinolones or tetracyclines addition. The purpose of this case report is to highlight the importance of proper selection of antibiotics for treatment of severe MPP and increase awareness concerning the emergence of fluoroquinolone-resistant MPP.

**Patient concerns:**

A case of severe life-threatening pneumonia in a 26-year-old man with high fever and cough was non-responsive to azithromycin and fluoroquinolones.

**Diagnoses:**

The patient was diagnosed with MPP based on the test results of bronchoalveolar lavage using real-time quantitative PCR method.

**Interventions:**

Tigecycline was given to the patient after azithromycin and fluoroquinolones failed.

**Outcomes:**

The patients fever subsided within the first day of tigecycline therapy. He showed rapid symptom resolution and improvement in lung infiltration after 4 days of tigecycline therapy.

**Lessons:**

The case suggests that fulminant MPP should be timely treated with proper antibiotics, and the possible emergence of fluoroquinolone-resistant MPP should be of concern.

## 1 Introduction

*Mycoplasma pneumoniae* (MP) is a common cause of community-acquired pneumonia. Most MP pneumonia (MPP) cases are mild to moderate, and severe cases are relatively rare with an incidence rate of 0.5% to 2%.^[1–3]^ Three major drug classes are used for treating MP infection: macrolides, quinolones, and tetracyclines. Currently, the MP macrolide resistance rate in China is as high as 97%.^[4]^ Clinical cases of quinolone and tetracycline resistance have not been reported. However, induction of quinolone resistance in MP has been shown in vitro.^[5]^ Adult MPP cases with macrolide treatment failure can be controlled quickly by switching to quinolones.^[6]^ However, herein, we describe a patient with severe MPP and azithromycin, levofloxacin, and moxifloxacin treatment failures, whose condition rapidly improved after switching to tigecycline.

Written informed consent was obtained from the patient for the use of the chest image data and the publication of this study.

## 2 Case report

A previously healthy 26-year-old man presented to a clinic with a 1-day history of high temperature of 39°C and non-productive cough on April 21, 2017. He received levofloxacin via infusion (0.6 g, once daily), but his symptoms did not improve after 5 days of therapy. On day 6, he experienced severe cough, accompanied by wheezing following exertion.

On day 7, blood testing at a local hospital revealed a lactate dehydrogenase (LDH) level of 450 U/L; chest computed tomography (CT) revealed consolidation in the left upper lung lobe. Subsequently, he received azithromycin infusion with methylprednisolone (40 mg, once daily) for 6 days. However, his fever persisted and the wheezing worsened; chest CT showed an expanded area of consolidation. On day 13, he was transferred to our hospital.

On admission, his vital signs were as follows: temperature, 39.0°C; respiratory rate, 25 breaths/minute; pulse, 130 beats/minute; and blood pressure, 125/80 mm Hg; left basilar rhonchi were noted. Laboratory evaluation showed the following: white blood cell count, 8.18 × 10^9^/L; neutrophils, 70.4%; C-reactive protein level, 156 mg/L; and LDH level, 371 U/L. Arterial blood gas analysis revealed an oxygen partial pressure of 59 mm Hg while breathing ambient air. Following admission, his body temperature increased to 40.0°C, and oxygen saturation decreased continuously, despite receiving meropenem (1 g, q8 h) and moxifloxacin (400 mg once daily) for 3 days. No bacteria or fungi were detected from the culture of respiratory samples collected at admission. Serum IgM antibody test results for influenza virus, adenovirus, respiratory syncytial virus, coronavirus, metapneumovirus, *Mycoplasma pneumoniae*, *Chlamydia,* and *Legionella pneumophila* were negative. Real-time quantitative PCR method was used to detect influenza virus and MP from throat swabs, and the results were negative.

**Figure 1:**
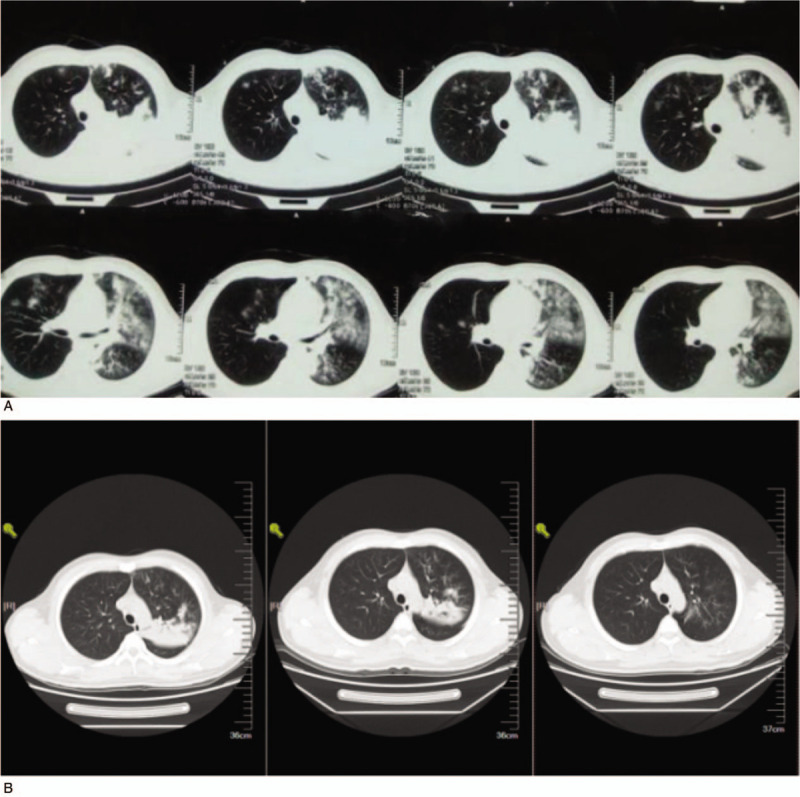
Chest computed tomography (CT) findings. (a) CT scan from May 2, 2017, showing consolidation in the left superior lobe and ground-glass opacification in both superior lobes. (b) CT scan from May 9, May 15, and June 20, showing gradual resolution of the consolidation in the left superior lobe and resolution of ground-glass opacification in both superior lobes.

Bronchoscopy was performed on hospitalization day 3. Bronchoalveolar lavage (BAL) fluid was screened for common respiratory pathogens using real-time quantitative PCR; no pathogen was identified, except MP (nucleic acid concentration, 2.4 × 10^8^ copies/ml). No bacteria or fungi were detected in the BAL fluid culture. Owing to azithromycin and fluoroquinolone treatment failure, tigecycline was administered on hospitalization day 4. His fever subsided within 24 hours. After 4 days of tigecycline therapy, we noted rapid symptom resolution and improvement in lung infiltration (Fig. 1). Oxygen partial pressure increased from 59 mm Hg to 81 mm Hg while breathing ambient air. MP nucleic acid concentration in BAL decreased from 2.4 × 10^8^ copies/ml (day 3) to 3.0 × 10^4^ copies/ml (day 10). Paired serology, with samples collected 10 days apart (on days 1 and 10), showed that anti-MP IgM had changed from negative to positive (1:640).

After discharge, the patient received minocycline for 10 days. During the 1-month follow-up visit after discharge, he showed no symptoms, and chest CT performed 21 days after discharge revealed limited features of bronchiolitis in the left lung (Fig. 1).

Sequencing of MP 23S rRNA in BAL fluid was performed. An A-to-G transition at nucleotide 2066 was found. High-throughput sequencing of MP DNA was performed to identify the presence of quinolone-resistant genes or mutation sites; however, the results were negative for both.

## 3 Discussion

In this case report, we describe a severe life-threatening case of MP pneumonia. Initial therapy involved administration of levofloxacin, azithromycin with corticosteroids, and moxifloxacin, but all these medications proved ineffective. However, following initiation of tigecycline administration, fever subsided within 24 hours, with rapid resolution of the respiratory failure symptoms and pulmonary infiltrates.

We diagnosed the patient with MPP based on the patients clinical course, CT manifestations, the shift in paired serum IgM against MP from negative to positive, repeated negative results for bacterial culture tests from respiratory tract specimens, and high MP DNA concentration detected by real-time PCR.

MP infection is generally self-limiting and rarely fatal; however, 0.5% to 2% of patients with MPP develop severe, life-threatening illnesses, and some patients experience complete respiratory failure and acute respiratory distress syndrome.^[1–3]^ Antimicrobial agents for the treatment of MP infections include macrolides, quinolones, and tetracyclines. For many years, macrolides were considered as first-line therapy for MP infections. However, macrolide resistance has been spreading worldwide for 20 years, with a prevalence of 90% to 100% in Asia. The major cause of macrolide resistance in MP is the loss of macrolide binding to the 23S rRNA components of MP ribosome due to a mutation at the target site in domain V of the 23S rRNA. The most frequently found basis of resistance is an A-to-G mutation at position 2063 at this site (A2063G), followed by A2063T, A2064G, and A2063C.^[6]^ MPP cases exhibiting resistance to macrolides could involve more severe clinical outcomes, such as a long fever duration, cough, or hospital stay. Multiple alternative antibiotic treatments might be required to treat this condition. Moreover, delayed administration of adequate antibiotics may contribute to the severity of MPP. Azithromycin infusion with the steroid methylprednisolone (40 mg, once a day) was administered to our patient for 6 days, but his symptoms continued to worsen. We sequenced the 23S rRNA of MP DNA extracted from BAL and found a rare and novel A-to-G point mutation at the 2066 locus, which has not been reported before. Because the nucleotide 2066 is part of a codon that encodes an important binding site for azithromycin, we surmised that this mutation might be the main reason for azithromycin treatment failure.

Currently, no fluoroquinolone-resistant MP clinical isolates have been reported. However, fluoroquinolone-resistant strains have been selected in vitro, and the mechanism of resistance was found to be mutations within the conserved regions of the *gyrA, gyrB, parC*, and *parE* genes, referred to as the quinolone resistance-determining regions.^[5]^ In this case, the patient was treated with levofloxacin for 6 days and moxifloxacin for 3 days, without any improvement. We performed high-throughput sequencing to identify any mutation sites or quinolone-resistant genes. However, we found no mutations (by Basic Local Alignment Search Tool [BLAST] comparison) or quinolone resistance genes (compared using the drug resistance library). We suspect that the increase in MIC values for fluoroquinolones in response to MP infection may have led to treatment failure. The epidemiological surveillance of fluoroquinolone resistance is of great interest for early detection and for identification of fluoroquinolone-resistant clinical isolates associated with the increasing use of this class of antibiotic.

Tigecycline is a 9-t-butylglycylamido derivative of minocycline; this novel antibiotic inhibits protein synthesis. It has broad-spectrum activity against a wide range of microorganisms, including both gram-positive and gram-negative bacteria. Moreover, tigecycline has shown efficacy in vitro and in vivo against MP. Tigecycline treatment has been demonstrated to be effective against MP, significantly improves histological lung inflammation and can reduce both pulmonary cytokine and chemokine levels_._^[7]^ To et al reported that fulminant macrolide-resistant MPP was exacerbated despite azithromycin administration, but marked improvement was noted after tigecycline administration.^[8]^ Similar to that adult case, our patient with severe macrolide-resistant MPP also showed rapid improvement after switching to tigecycline.

Diagnosis of MP infections can be challenging, as these bacteria are not visible by Gram staining due to the lack of a cell wall. Current diagnostic modalities include various direct PCR assays, serology, and culture. The sensitivity of a PCR assay depends on both the technique and sample. In this case, real-time quantitative PCR using throat swab samples showed negative results for MP, whereas that of BAL sample yielded strongly positive results for MP. These results demonstrate that BAL might be better than nasopharyngeal or oropharyngeal swabs for detection of MP in patients with MPP. The gold standard for serological diagnosis of MPP is a four-fold change in antibody titers over time. However, in this case, the first serological test result for anti-MP IgM, conducted on day 11 of onset, was negative. In the early stages of MPP, serologic testing could have some limitations because of the absence of IgM antibody responses against MP, especially in severe cases. Therefore, repeated IgM or IgG tests are required for early confirmative diagnosis during hospitalization. MP culturing is time-consuming and not readily available in most labs.

To the best of our knowledge, the present report is the first documented case of severe life-threatening MPP due to a macrolide-resistant strain that was non-responsive to azithromycin with corticosteroids, levofloxacin and moxifloxacin. Based on the experience gained from the present case, patients with fulminant MPP (due to this macrolide-resistant strain) should receive other classes of antibiotics within 48 to 72 hours of failure of initial treatment. Tigecycline was effective against MPP in the present case, and its use should be considered in future clinical trials.

## 4 Conclusion

We report a rare case of near-fatal MPP due to a macrolide-resistant strain that was non-responsive to both macrolides and fluoroquinolones. This case highlighted the importance of proper selection of antibiotics for treatment of severe MPP, and may contribute to increased awareness concerning the emergence of fluoroquinolone-resistant MPP infections, particularly in cases where initial treatment fails.

## Acknowledgments

We would like to express our warmest appreciation to FengJ Li and Shun Meng for their assistance in collecting the specimen.

## Author contributions

**Data curation:** Xin Yuan.

**Formal analysis:** Xin Yuan.

**Funding acquisition:** Changqing Bai.

**Investigation:** Xin Yuan.

**Methodology:** Xin Yuan, Changqing Bai.

**Writing – original draft:** Xin Yuan.

**Writing – review & editing:** Xin Yuan, Changqing Bai, Yuan Luo.
